# Racial, ethnic, and rural disparities in access to Medicaid offices

**DOI:** 10.1093/haschl/qxaf072

**Published:** 2025-04-04

**Authors:** Kenneth Lim, Demetri Goutos, Monica Aswani, Joseph Benitez, Kathryn Thompson, Paul R Shafer

**Affiliations:** Department of Health Law, Policy, and Management, School of Public Health, Boston University, Boston, MA 02118, United States; Department of Health Law, Policy, and Management, School of Public Health, Boston University, Boston, MA 02118, United States; Department of Health Services Administration, School of Health Professions, University of Alabama at Birmingham, Birmingham, AL 35294, United States; Martin School of Public Policy and Administration, University of Kentucky, Lexington, KY 40506, United States; Department of Community Health Sciences, School of Public Health, Boston University, Boston, MA 02118, United States; Department of Health Law, Policy, and Management, School of Public Health, Boston University, Boston, MA 02118, United States

**Keywords:** Medicaid offices, enrollment support, disparities

## Abstract

Physical Medicaid offices may play an important role in supporting beneficiaries and alleviating administrative burdens during critical enrollment or redetermination periods. Limited research has explored whether racial, ethnic, and rural disparities in access to Medicaid offices exist. Leveraging a county-level data set of geocoded Medicaid offices merged with American Community Survey data, we examined variation in Medicaid office density. We used a choropleth map to demonstrate variability within and across states and linear probability models to explore the association between demographic characteristics and having at least 1 Medicaid office in the county. Over three-fourths of US counties had an office, but access to such offices varied by race, ethnicity, and rurality. Counties with high Hispanic (≥40%) and rural (>50%) populations were associated with a 14.3 and 18.7 percentage point lower probability of having at least 1 Medicaid office (both *P* < 0.001), respectively. Findings can be used to prioritize areas for investment in physical infrastructure, specific group outreach, or technological advancements by state Medicaid programs. While the unwinding from the COVID-19 public health emergency may have highlighted these vulnerabilities and inequities, our findings reflect long-standing differences in investment across states that influence individuals’ access to Medicaid.

## Teaser

Over three-fourths of US counties had at least 1 Medicaid office, but access varied by race, ethnicity, and rurality. Counties with high Hispanic and rural populations had a lower probability of having a Medicaid office. To facilitate smooth enrollment processes and reduce administrative burdens, state Medicaid programs may consider investments into additional physical offices, community-based organizations for specific group outreach, or further advancements in technological processes such as online enrollment systems or mobile services.

## Introduction

Medicaid, which insures approximately 21% of individuals and finances 41% of births,^1,2^ is an integral component of the US healthcare system. Its extensive reach is primarily the result of the Affordable Care Act of 2010, which expanded Medicaid eligibility to include a greater share of low-income Americans.^3^ Considerable research has documented the positive impacts of Medicaid expansion on health and financial outcomes, healthcare affordability, access to medical care, and self-reported physical and mental health.^4-6^ During the COVID-19 public health emergency (PHE), the continuous enrollment provision contributed to the growth of the Medicaid program to nearly 95 million enrollees, without the typical administrative burdens associated with remaining enrolled.^7^

Administrative burdens are complex, often unclear processes or requirements that create barriers to enrolling or re-enrolling in safety-net programs such as Medicaid, disproportionately impacting communities of color.^8,9^ These burdens include the time and effort needed to learn about program benefits and eligibility, navigate application or reapplication processes, and comply with bureaucratic documentation requirements.^10^ Research has shown that administrative burdens contribute to individuals losing Medicaid coverage or churning on and off coverage, which may interrupt access to care and lead to poor health outcomes.^8,11^ As of September 2024, approximately 25 million individuals have lost Medicaid coverage since the unwinding began in March 2023, 69% of which have had their coverage terminated for procedural reasons (eg, not completing renewal process due to paperwork, outdated contact information), despite potentially still being eligible.^12^ Increasing access to enrollment support is 1 approach to reducing the negative impacts of administrative burdens.

Because Medicaid is federally supported but state-administered, there is considerable variation in program design and implementation. We investigate 1 area of enrollment support that has not been well-documented in the literature—Medicaid offices—in an effort to understand whether administrative burdens are exacerbated through underinvestment in offices and staff necessary to support the program and its beneficiaries. Allocation of Medicaid offices varies by state, as offices may be independent or housed within larger governmental administration buildings (eg, Illinois Department of Human Services offices serve Medicaid and other public benefit programs), and offices are financed through a myriad of state and federal resources (eg, state taxes, 50% Medicaid federal matching rate for administrative costs).^13,14^ Despite a shift toward online enrollment platforms, physical access to Medicaid offices serves as a complement to support enrollment and retention in the program rather than a substitute. Medicaid offices serve important roles in assisting beneficiaries with understanding eligibility requirements, filling out applications, and navigating complex documentation processes.^15^ Though research on the benefits of physical Medicaid offices is limited, with none to our knowledge documenting the potentially shifting modality of enrollment over time (eg, office vs online), they still serve a critical function alongside online options, in a similar way to how customer service in other industries operates both in-person and digital offerings. Using a novel, geocoded data set of public-facing Medicaid office locations, we describe county-level variation in access to offices and investigate whether racial, ethnic, and rural disparities exist.

## Methods

### Data sources

To examine the relationship between population demographics and Medicaid office access, we relied on 2 primary data sources. First, we utilized a hand-collected, open access database of geocoded public-facing Medicaid office locations as of late 2023.^15^ These data focused on Medicaid offices that provide services to the public, such as enrollment support and access to caseworkers, excluding those that only provide client services (eg, behavioral health, home and community-based services for long-term care needs) or house administrative functions (eg, application or claims processing). Second, we obtained American Community Survey (ACS) 5-year estimates (2017-2021) from the US Census Bureau to describe county-level population and demographics including race, ethnicity, rurality, and income.^16^ We assigned each office to its county location based on latitude and longitude coordinates provided in the source data using the Census Block Conversions Applications Programming Interface^17^ in R and then collapsed the data to a county-level count of Medicaid offices present, from which we also developed an indicator for having a Medicaid office present in a given county. We then merged the Medicaid offices data with ACS data by state and county FIPS codes for analysis. The resulting data set was cross-sectional and organized at the county level.

### Analysis

**Figure 1.**
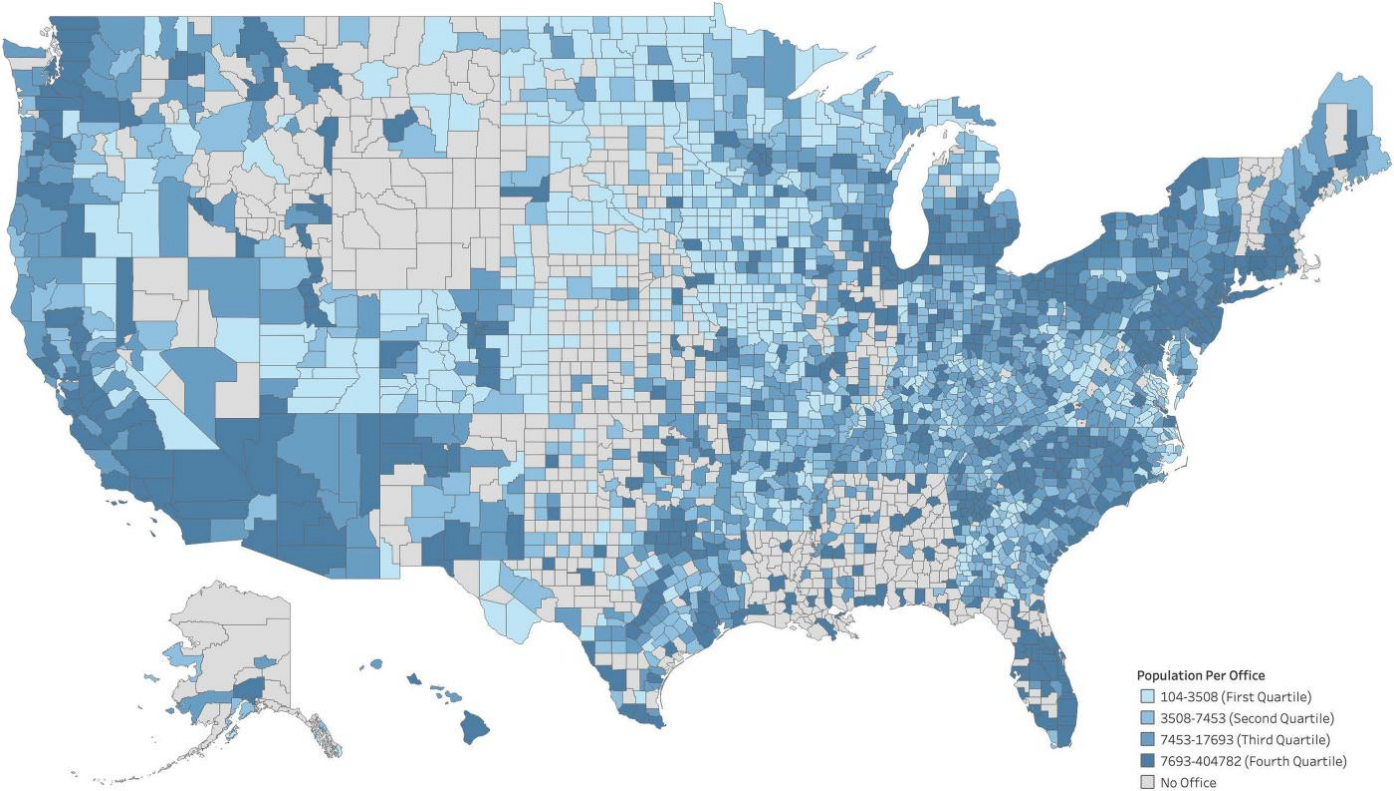
Population under 150% FPL served per Medicaid office. Source: Authors’ analysis of geocoded Medicaid offices database and publicly available American Community Survey data (5 Year Estimates from 2017-2021). FPL, federal poverty level. Legend indicates quartiles for the county population under 150% of the FPL that can be attributed to a single Medicaid office. For example, counties in darker blue have a greater concentration of Medicaid-eligible individuals for 1 office.

First, we examined variation in Medicaid office density descriptively, calculating the share of counties with an office present and county population under 150% of the federal poverty level (FPL) attributable to each Medicaid office, both nationally and visualized across all US counties using a choropleth map (Figure 1). Second, we used linear probability models to understand how density of racial and ethnic minorities, low-income residents, rurality, and Medicaid expansion status were associated with the probability of having at least 1 Medicaid office in a county, using our cross-sectional merged data set. We operationalized this by including indicator variables for 20% to <40% population non-white, ≥40% population non-white, 20% to <40% population Hispanic or Latino, ≥40% Hispanic or Latino, > 50% population living in rural block, state Medicaid expansion status (as of May 2024), and the population under 150% FPL for each county, building on previous work.^18^ In all models, we included state fixed effects to account for unobserved, time invariant state characteristics that may bias our results. We also included 2 sensitivity analyses. First, in addition to original predictors, we included the number of Medicaid managed care (MMC) plans in a state and federally qualified health centers (FQHCs) in a county, derived from Health Center Program Uniform Data System data for 2022, into our model. Second, we included an interaction between race and rurality to assess whether their compounded effect is associated with having a Medicaid office in the county.

### Limitations

Our study has a few limitations. First, we were unable to account for Medicaid office size, staff capacity, and geographical distance to an office, all of which may impact access to and level of enrollment support. We also could not account for other enrollment support sources outside of FQHCs or MMC plans—such as assistors, community-based organizations, call centers, or other online resources—that may be involved in helping individuals enroll in Medicaid. Second, the Medicaid offices data used were gathered during and following the COVID-19 PHE and may not reflect changes in access as offices were closed or combined as a result of the pandemic. Third, our analysis is purely associative and does not imply any causal relationship.

## Results

Over three-fourths of US counties (2409 of 3143, 76.6%) had at least 1 public-facing Medicaid office (ranging from 1 to 25), leaving nearly a quarter of counties that did not (23.4%). Among counties with at least 1 public-facing Medicaid office, the median population attributable was 7453 individuals under 150% FPL per office, with a range of 104 to 404 782 per office. There was considerable geographic variation across and within states in terms of presence of offices and population attributable to each office in counties where they are present (Figure 1).

Table 1 shows the results of linear probability models describing the association between demographics and other characteristics of counties to the presence of at least 1 public-facing Medicaid office (county characteristics based on office status are included in Appendix A). We found that counties with high non-white populations (≥40%) were associated with a 7.5 percentage point higher probability of having at least 1 Medicaid office (*P* < 0.001). Counties with high Hispanic or Latino populations (≥40%) were associated with a 14.3 percentage point lower probability of having at least 1 Medicaid office (*P* < 0.001). Counties with a majority of their population living in rural Census blocks were associated with an 18.7 percentage point lower probability of having at least 1 Medicaid office (*P* < 0.001). Counties located in a Medicaid expansion state were associated with an 88.2 percentage point higher probability of having at least 1 Medicaid office (*P* < 0.001). The sensitivity analyses yielded similar coefficient estimates with the added measures—the number of MMC plans in a state and FQHCs in a county (Model 2) and the race and rurality interaction (Model 3)—providing little additional explanatory value.

**Table 1.** Linear regression results for probability of having a Medicaid office present in a county.

	Main analysis	Sensitivity analyses	
Variables	(1)	(2)	(3)
20 to <40% non-white	9.500***	9.502***	9.542***
	(1.819)	(1.820)	(1.827)
≥40% non-white	7.454**	7.475**	8.056*
	(2.398)	(2.401)	(3.245)
20 to <40% Hispanic/Latino	−4.508	−4.510	−4.557
	(2.912)	(2.913)	(2.921)
≥40% Hispanic/Latino	−14.34**	−14.31**	−14.40**
	(4.390)	(4.402)	(4.398)
Rural	−18.72***	−18.74***	−18.61***
	(1.298)	(1.297)	(1.341)
20 to <40% under 150% FPL	1.571	1.538	1.631
	(1.724)	(1.726)	(1.749)
≥40% under 150% FPL	20.47	20.46	20.75
	(17.34)	(17.38)	(17.47)
Medicaid expansion status	88.16***	70.38***	88.10***
	(4.685)	(4.024)	(4.710)
Population under 150% FPL	1.93**	1.64	1.89*
	(0.74)	(0.916)	(0.753)
Number of FQHCs in county	N/A	0.229	N/A
		(0.766)	
Number of MMC plans in state	N/A	5.89	N/A
		(0.28)	
≥40% non-white and rural interaction	N/A	N/A	−1.031
			(3.749)
Constant	23.29***	23.81***	23.26***
	(4.382)	(4.385)	(4.361)
Observations	3143	3143	3143
*R* ^2^	0.503	0.503	0.503

Source: Authors’ analysis of geocoded Medicaid offices database and publicly available American Community Survey data (5 Year Estimates from 2017-2021). *P < 0.05, **P < 0.01, ***P < 0.001. This is a cross-sectional analysis using linear probability models. The unit of analysis is the county. Coefficients and standard errors (in parentheses) are expressed as percentage points. The “population under 150% FPL” variable was transformed to *Z* scores in regression and can be interpreted in terms of standard deviations from the mean.

Abbreviations: FQHC, federally qualified health center; MMC, Medicaid managed care.

## Discussion

Using a novel data set of public-facing Medicaid office locations and county-level ACS data, we examined the geographic distribution of Medicaid offices in the United States and their association with county demographics. We found that despite three-fourths of US counties having a Medicaid office, access to offices varied as counties with larger proportions of Hispanic/Latino and rural populations had a lower probability of having a Medicaid office.

To our knowledge, this is the first study to investigate geographical variation of and disparities in access to public Medicaid offices. Most of the Medicaid literature focuses on how access to care is influenced by insurance eligibility criteria, provider capacity constraints, low income or poor health status, or being a member of a minority group,^19-21^ but not on the role of Medicaid offices as a potential mechanism for helping enrollees navigate burdensome enrollment policies and procedures. Our work provides insights into a key, yet often overlooked, element of enrollment assistance that has important considerations for patient access to care and potential areas for programmatic improvements.

There are several potential explanations for our results. First, population density likely influences states’ investment decisions. Since rural counties have lower population densities than urban areas, states may prioritize the latter as it may be more efficient and cost-effective to focus on areas with higher concentration of potential enrollees. This coincides with historical underinvestment in and marginalization of rural communities across federal programs.^22^ Second, Hispanic populations may require additional resources, such as multilingual staff, interpreters, or cultural competency programs, which may deter investment. These barriers reflect broader patterns of structural racism and underinvestment in Hispanic communities.^23,24^

However, it may also be likely that as many states have shifted toward digital platforms to support Medicaid enrollment processes, state variation in online and phone-based enrollment options, as well as the extent to which such options are made available, may influence decisions on whether to invest in Medicaid offices. For example, states with strong technology to support Medicaid enrollment may not view physical office infrastructure as being entirely necessary if their current approaches are sufficient to maintain enrollment levels. Despite this, because Medicaid offices can complement other enrollment mechanisms and support beneficiaries, our findings remain valuable as they illustrate important disparities that can influence health insurance coverage for many Americans.

Our results have potential policy implications for state Medicaid programs. First, state Medicaid agencies might consider opening additional public-facing offices in counties with predominantly rural and Hispanic/Latino populations, or counties with a greater Medicaid-eligible population attributable to a single office, to assist with enrollment assistance if they believe such investments would be beneficial and cost-effective. However, this option may be difficult as certain areas, such as rural counties, may not have the population to support a Medicaid office and many state Medicaid programs experience budgetary issues. To circumvent this, a second, more viable policy solution may be for states to partner with community-based organizations to provide more focused and culturally competent outreach to these groups and serve as a resource for enrollment and eligibility. Third, states could consider enhancing their online enrollment systems and expanding their mobile enrollment services to underserved communities, which may reduce the need for in-person assistance and support.

## Conclusion

Administrative burdens have led millions of individuals to be kicked off Medicaid, despite still being eligible for the program and the numerous benefits it offers. Medicaid offices may play a vital role in helping beneficiaries navigate the program's complex eligibility and enrollment requirements, serving as a key resource in reducing the impact of such administrative burdens. Our analysis found that despite over three-fourths of US counties having a Medicaid office, access to these offices varies considerably across racial, ethnic, and rural groups, providing potential opportunities for state investment. States should consider various strategies to support rural and Hispanic/Latino beneficiaries to ensure that they maintain coverage and have access to comprehensive health care.

## Supplementary Material

qxaf072_Supplementary_Data
