# Supplementary material for: Racial, ethnic, and rural disparities in access to Medicaid offices
Source: Health Aff Sch. 2025 Apr 4;3(5):qxaf072. doi: 10.1093/haschl/qxaf072 (PMC12053252; doi:10.1093/haschl/qxaf072)
Supplement: qxaf072_Supplementary_Data [file qxaf072_supplementary_data.zip › Appendix A.docx]

|  | **Total** | **No Office** | **Has Office** | **P-value** |
| --- | --- | --- | --- | --- |
| Number of US Counties | 3,143 | 734 | 2,409 |  |
| **Main County Characteristics (%)** | | | | |
| Race/Ethnicity |  |  |  |  |
| 40%+ Non-White | 12% | 14% | 12% | 0.16 |
| 40%+ Hispanic/Latino | 5% | 7% | 5% | 0.008 |
| Rural | 64% | 85% | 58% | <0.001 |
| Medicaid Expansion Status | 69% | 55% | 73% | <0.001 |
| US Region |  |  |  | <0.001 |
| Midwest | 34% | 29% | 35% |  |
| Northeast | 7% | 4% | 8% |  |
| South | 45% | 48% | 44% |  |
| West | 14% | 19% | 13% |  |
| **Other County Characteristics (mean, SD)** | | | | |
| Age |  |  |  |  |
| % 19 and Under | 0.25 (0.04) | 0.25 (0.04) | 0.25 (0.04) | 0.12 |
| % 20 to 29 | 0.12 (0.03) | 0.11 (0.03) | 0.12 (0.03) | <0.001 |
| % 30 to 39 | 0.12 (0.02) | 0.12 (0.02) | 0.12 (0.02) | <0.001 |
| % 40 to 49 | 0.12 (0.02) | 0.11 (0.02) | 0.12 (0.01) | <0.001 |
| % 50 to 65 | 0.21 (0.03) | 0.21 (0.03) | 0.20 (0.02) | 0.004 |
| % 65 and Older | 0.19 (0.05) | 0.20 (0.05) | 0.19 (0.05) | <0.001 |
| Sex |  |  |  |  |
| % Female | 0.50 (0.02) | 0.49 (0.03) | 0.50 (0.02) | <0.001 |
| Average Household Income | 76,444 (19,299) | 73,276 (16921) | 77,409 (19870) | <0.001 |
| % Population Insured | 0.90 (0.05) | 0.89 (0.05) | 0.91 (0.05) | <0.001 |
| % Under FPL | 0.14 (0.06) | 0.14 (0.07) | 0.14 (0.06) | 0.54 |
| % with Disability | 0.16 (0.05) | 0.17 (0.05) | 0.16 (0.05) | <0.001 |

**Appendix A. County Characteristics by Medicaid Office Status**

**Source**: Authors' analysis of geocoded Medicaid offices database and publicly available American Community Survey data (5 Year Estimates from 2017-2021)

**Notes**: Main county characteristics were included as explanatory variables in our models. Other county characteristics are included for additional context. For main characteristics, an example interpretation is: "14% of counties with no Medicaid office were 40% or more non-white." For other county characteristics, an example interpretation is: "The mean percent of individuals 19 and under in counties with no Medicaid office was 25%." Other county characteristics, which are continuous, reflect county-level averages, where characteristics were collected for each county (e.g., proportion of individuals who are insured, average household income), then averaged across counties.
